# Fresh-blood-free diet for rearing malaria mosquito vectors

**DOI:** 10.1038/s41598-018-35886-3

**Published:** 2018-12-13

**Authors:** Joana Marques, João C. R. Cardoso, Rute C. Felix, Rosa A. G. Santana, Maria das Graças Barbosa Guerra, Deborah Power, Henrique Silveira

**Affiliations:** 1 Global Health and Tropical Medicine, GHTM, Instituto de Higiene e Medicina Tropical, IHMT, Universidade Nova de Lisboa, UNL, Rua da Junqueira 100, 1349-008 Lisboa, Portugal; 2 Comparative Endocrinology and Integrative Biology, Centre of Marine Sciences, Universidade do Algarve, Campus de Gambelas, 8005-139 Faro, Portugal; 3 Fundação de Medicina Tropical Dr. Heitor Vieira Dourado, Manaus, AM Brazil; 4 Universidade do Estado do Amazonas, Manaus, AM Brazil

## Abstract

Mosquito breeding depends on the supply of fresh vertebrate blood, a major bottleneck for large-scale production of *Anopheles* spp. Feeding alternatives to fresh blood are thus a priority for research, outdoor large-cage trials and control interventions. Several artificial meal compositions were tested and *Anopheles* oogenesis, egg laying and development into the next generation of adult mosquitoes were followed. We identified blood-substitute-diets that supported ovarian development, egg maturation and fertility as well as, low progeny larval mortality, and normal development of offspring into adult mosquitoes. The formulated diet is an effective artificial meal, free of fresh blood that mimics a vertebrate blood meal and represents an important advance for the sustainability of *Anopheles* mosquito rearing in captivity.

## Introduction

Malaria elimination and the current rise of other vector-borne diseases has led to the development of several innovative mosquito control methods^1–3^ that largely depend on the release into the wild of genetically modified mosquitoes produced in captivity. Most vector mosquitoes are anautogenous which means that females require a vertebrate blood meal for egg production and development^4^. A major bottleneck for establishing effective mosquito breeding in captivity is that production methods depend on a supply of blood from different animal sources and its quality is often variable. Current mosquito laboratory rearing strategies use as blood sources sedated or restrained live animals (mice, rats, chickens) or human blood. Nevertheless, foreseeing the urgent need to produce mosquitoes on a large scale, the use of large quantities of blood constitutes a drawback due to ethical concerns and logistical issues associated with demanding safety regulations. In addition, the mass rearing of mosquitoes requires a specialized animal care facility, qualified personnel, and an efficient feeding system. These facts might result in a significant hindrance since local regulations, ethical concerns, and infrastructure vary considerably between different countries. Taken together, these limitations are prompting research directed at the development of an artificial meal capable of mimicking blood in terms of producing viable mosquito eggs.

Artificial diets based on the rich nutrient content of human blood have been shown to prompt female mosquito oogenesis and fertility *in vivo* with limited success in comparison to a standard vertebrate blood meal^5–8^. Successful artificial diets for vector mosquitoes, must fulfil the following requirements: (1) female mosquitoes must fully engorge when feeding, (2) the artificial diet must allow vitellogenesis to occur, (3) it must result in large egg batches, and (4) the offspring fitness should be comparable to wild mosquitoes^9^. Until now, little is known about artificial meals for *Anopheles* spp. but several studies have been published for *Aedes* mosquitoes. Alternative vertebrate blood-free meals for *Aedes* mosquitoes must supply an energy source for mosquitoes to feed (e.g. ATP^9^), a protein source essential for egg maturation^5,10^, carbohydrates for energy consumption, and amino acids (aa)^11^ that are vital for egg production. In fact, the aa composition is one of the major limiting factors of female mosquito fertility as this depends on the blood source^12^. Besides proteins, a blood meal might also provide lipids, particularly cholesterol^13^ for egg production improvement. All of these components are also probably necessary for meals targeted to *Anopheles* mosquitoes.

Insect reproduction and nutrient metabolism are also regulated by a series of neuropeptides many of which act through the activation of heterotrimeric GTP-binding protein (G protein)–coupled receptors in the presence of an internal or external stimuli^14^. GPCRs are a large family of cell surface proteins. At least 276 receptor genes exist in the *Anopheles* genome, many of which are orphans and have currently uncharacterised functions^15^. Conserved sequence homologues of mosquito GPCRs exist in humans and their activating peptides circulate freely in blood and regulate a diversity of physiological functions including energy metabolism and reproduction. This means that during mosquito blood feeding it is likely that interactions between mosquito GPCRs and putative vertebrate peptides may occur.

The insect and human GPCR systems have recently been characterised using comparative genomics approaches^16,17^. The mosquito genome contains orphan receptor genes that have a similar sequence and may be functional homologues of some vertebrate GPCRs. The exposure of mosquitoes to human GPCR-activating peptides when they take a blood-meal raises interesting questions about co-evolution of protein-protein interactions and cross-regulation of physiological systems. In the present study we tested the hypothesis that human GPCR ligands in blood, potentially by binding to mosquito GPCRs, can stimulate egg development and viability when they take a blood meal. To test this hypothesis we formulated an artificial blood free meal for female *Anopheles* mosquitoes and supplemented it with physiological concentrations of several different human GPCR ligands. The effect of the formulated diet on egg development, fecundity and egg viability was compared with the vertebrate fresh blood meal. The study highlights the potential of the largely neglected field of co-regulatory mechanisms of host – vector physiology.

## Results and Discussion

### Selection of vertebrate peptide candidates

Candidate molecules that circulate in human blood plasma and that stimulate different GPCRs were selected based on, a) their involved in the regulation of reproduction and nutrient metabolism in humans and b) the identification of orphan GPCRs in the mosquito genome that are sequence orthologues of human receptors (Table S1). Human ligands for GPCRs of several receptor families were selected and included peptides that activate Class A GPCRs (a.k.a Rhodopsin family GPCRs): oxytocin, galanin (GAL), kisspeptin, neuropeptide Y (NPY) that are basic determinants of reproductive functions^18^, luteinizing hormone releasing hormone (LHRH), which stimulates the release of luteinizing hormone (LH) and follicle-stimulating hormone (FSH) and triggers ovulation in females^19^ and melatonin (MT) which regulates circadian rhythms of feeding^20^ and peaks in the blood at dawn (when mosquitoes are more actively searching a blood meal); Ligands of Class B GPCRs (a.k.a. Secretin-GPCRs) glucagon-like peptide 1 (GLP1), glucagon-like peptide 2 (GLP2) and vasoactive intestinal peptide (VIP) that regulate feeding behaviour^21^, gut motility^22–24^, glucose and insulin metabolism^25,26^ and parathyroid hormone (PTH), calcitonin (CT) and corticotrophin releasing hormone (CRH) which regulate ion metabolism and the stress response in humans and other vertebrates and also indirectly affects feeding behaviour^27–31^; and of Class C GPCRs (a.k.a Glutamate GPCRs) the Glutamate and ɣ-aminobutyric acid (or GABA) which are two important neurotransmitters that stimulate feeding^32^. Two fish peptides (CT and LHRH) were also tested as they are potent activators of the human peptide receptors and have similar functions to their human orthologues^33,34^. No potential activity or physiological effect of the selected human GPCR-ligands in mosquitoes is known.

**Figure 1:**
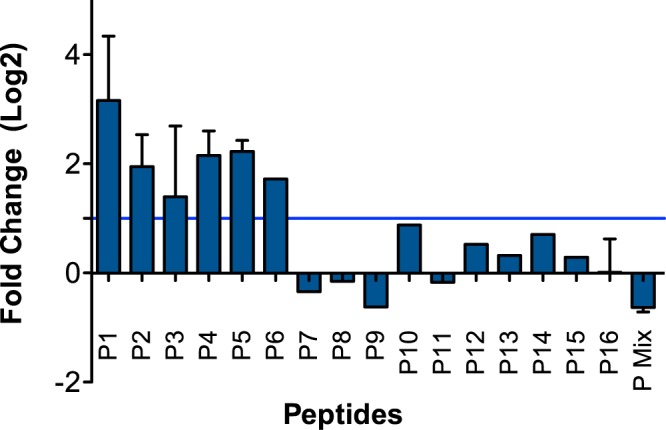
Relative expression of vitellogenin precursor 24 hours post-peptide injection. The results represent the mean ± SE of 3 biological replicates from 3 independent experiments. Relative expression of Vg was determined using the ΔΔCT method using as the control female mosquitoes injected with PBS.

Potential activity of human GPCR ligands in mosquitoes were initially screened *in vivo* for their capacity to trigger vitellogenin (Vg) transcription, a molecular marker of oogenesis initiation in the female mosquito fatbody^35^ (for peptide description see Supplementary Information, Table S1). Vg transcript abundance was evaluated 24 hours post-feed, when maximal Vg expression is expected (Supplementary Information, Fig. S1). Of the 16 vertebrate peptides tested and injected into the thorax of *Anopheles coluzzii* (previously known as *Anopheles gambiae* s.s. M form) only 6 (P1 to P6) induced up-regulation of Vg expression 24 h post-injection (Fig. 1) and their physiological effect on mosquito reproduction and fertility was subsequently tested.

### Formulation of the artificial fresh-blood-free meal

**Figure 2:**
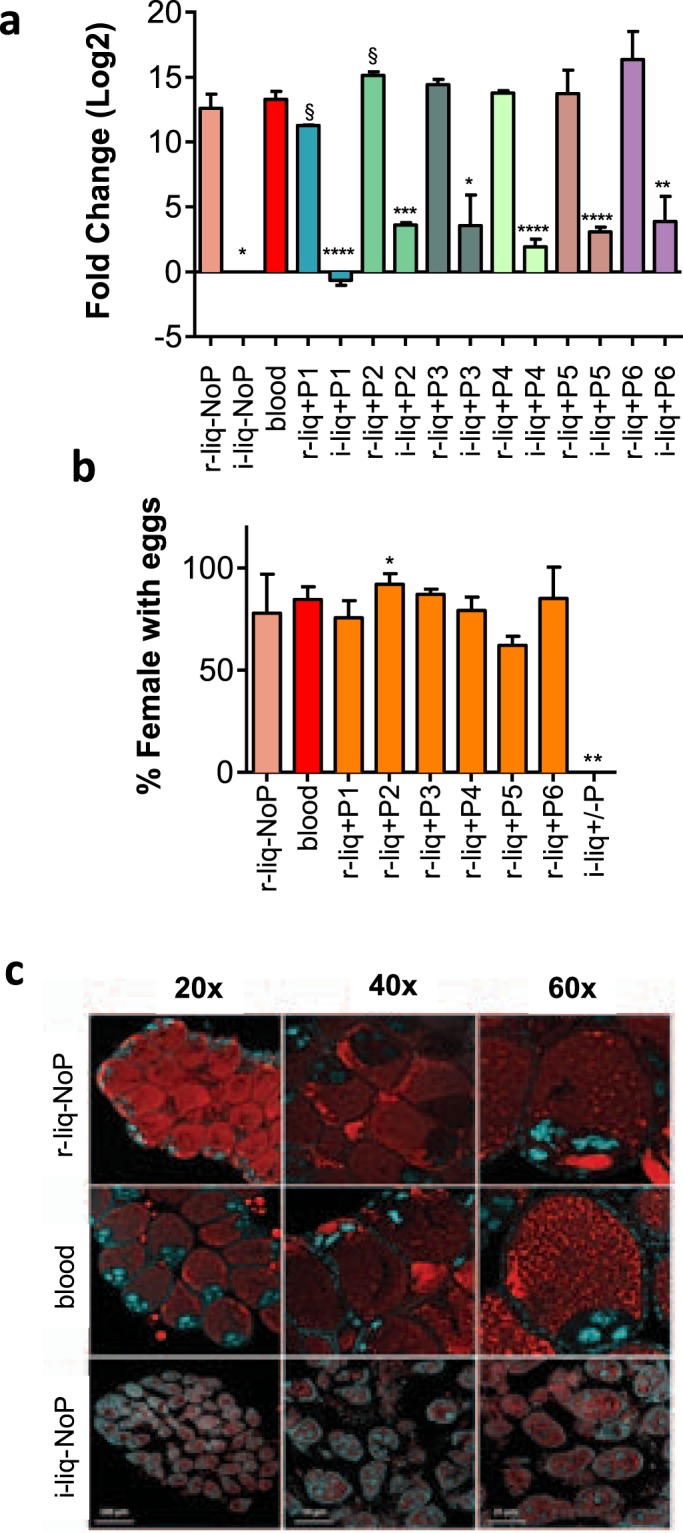
Effects of the fresh-blood-free diets on oogenesis. (**a**) Expression of vitellogenin precursor. Relative expression was determined using the ΔΔCT method as fold change to mosquitoes fed on the i-liq_diet. (**b**) Percent of mosquito females with eggs. Females were dissected 48 h post-feeding. The results represent the mean ± SE of 3 biological replicates. (**c**) Oocyte structure. Fluorescent confocal microscopy of oocytes stained with Nile red (lipids stain, red) and Dapi (nuclei stain, blue). Images were acquired with 20x, 40x, and 60x objectives using a Leica TCS SP5 laser scanning confocal microscope. Asterisks indicate statistical significant (P < 0.05) results of an unpaired *t* test when compared to the blood-fed group (control).

To test the potential involvement of the selected peptides on mosquito reproduction and egg development, different diets were formulated. Peptides were added either to an initial liquid diet (i-liq_diet) providing amino acids, vitamins and carbohydrates (based on DMEM tissue culture medium ref. 12–604 F from Lonza) or to a rich liquid diet (r-liq_diet), that consisted of i-liq_diet supplemented with: a) a phagostimulant (ATP), b) proteins (BSA) essential for egg maturation and c) cholesterol (For composition see Supplementary Information, Table S3). Insects cannot synthesize cholesterol de novo and it is a precursor of the ecdysteroid hormone with a key role in yolk synthesis and egg maturation^36^. Diets containing different peptides were supplied to female mosquitoes using a standard artificial feeding apparatus^37^ and compared with fresh mouse blood. Oogenesis was assessed by changes in Vg expression 24 h post-feeding (Fig. 2a). To confirm that Vg expression induced oogenesis progression, the number of females presenting eggs was evaluated. The oocyte structure and lipid deposition were also evaluated by confocal microscopy. Except for the i-liq_diet, oocyte and ovary development of mosquitoes fed on an artificial fresh-blood-free meal or blood diets was similar (Fig. 2b), and no morphological changes in ovary structure were observed (Fig. 2c). The r-liq_diet, r-liq_diet-supplemented with peptides and blood diet triggered similar Vg transcript levels suggesting that vitellogenesis occurred at a comparable rate in mosquitoes fed either on artificial diets or fresh blood. Of the blood-fed mosquitoes 85% produced eggs. Egg development occurred in 78% of the females fed on r-liq_diet and the highest percent of females with eggs (92% and 87%) was obtained for r-liq_diet + P2 (hGLP2) (p = 0.042) and r-liq_diet + P3 (hPTH) (Fig. 2b). No egg development was observed with i-liq_diet (with or without peptide).

### Feeding rate and egg production

**Figure 3:**
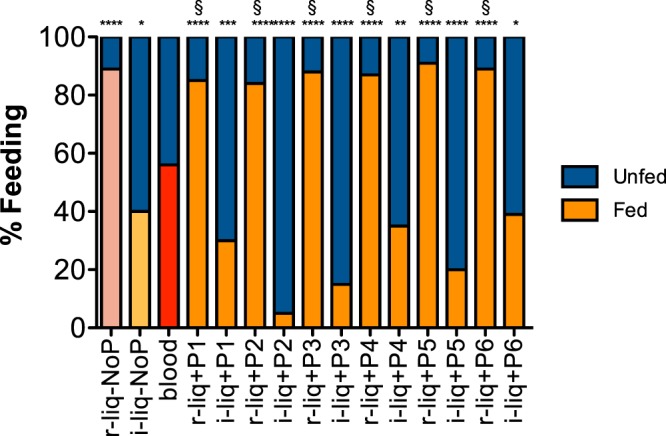
Feeding rate. The relative percent of fed and unfed females is indicated. Asterisks indicate groups significantly different (p < 0.05 to p < 0.0001) from the blood-fed control group and § indicates peptide supplemented r-liq groups significantly different (p < 0.0001) from the equivalent peptide enriched i-liq_diet (Fisher’s exact test). Blue: unfed; Salmon, Pale and Vivid Orange and Red: fed.

A successful artificial diet should be as attractive to female mosquitoes as a fresh blood meal. Therefore, mosquito feeding success was investigated. Groups of female mosquitoes were fed with the different diets and the proportion of fully fed females compared with the control blood-fed group. The percent of engorged female mosquitoes was always significantly higher in the r-liq_diet relative to the blood-fed group (Fig. 3). Approximately 60% of the blood-fed females were fully engorged relative to 40% of the mosquitoes fed with the i-liq_diet. The highest rate of feeding success, as assessed by the numbers of engorged females, was obtained for the r-liq_diet (84–91%). The highest rate of engorged females was obtained with the r-liq + P5 (hCT) diet (91.2% ± 6.8). Rates of engorgement with the r-liq_diet increased more than 20% relative to the blood-fed mosquitoes, suggesting that artificial diets can be highly attractive to female mosquitoes when using artificial membrane feeding (AMF).

### Fecundity and fertility

The nutritional quality of each diet was evaluated by assessing the fecundity and fertility of female mosquitoes during the first gonotrophic cycle after artificial membrane feeding (AMF). By including fertility and fecundity as well as initiation of oogenesis it was possible to directly establish the effect of the diet on successful egg production or egg laying. Fully engorged mosquitoes were maintained under optimal conditions (26 ± 1 °C, 75% humidity and a 12 h:12 h light:dark cycle) and egg laying observed 48 hours post-feeding. The egg number was counted 72 hours post-feeding (Table 1) before they were flooded with distilled water.

**Table 1:** Egg batches produced by *A*. *coluzzii* females.

	Total Egg Number (±SE)	Eggs/Female (±SE)
blood	733 ± 330	24 ± 11
r-liq_diet - NoP	763 ± 164	25 ± 5
r-liq_diet + P1	774 ± 343	26 ± 11
r-liq_diet + P2	648 ± 58	22 ± 1
r-liq_diet + P3	719 ± 82	25 ± 2
r-liq_diet + P4	492 ± 62	16 ± 2
r-liq_diet + P5	309 ± 18	10 ± 0
r-liq_diet + P6	656 ± 57	22 ± 2

The number of laid eggs was established using a hand-held magnifying glass. For each experimental diet three independent experiments were performed consisting of 30 female mosquitoes each (n = 90/diet). Consult supplementary information for peptide description.

Females that were fed on fresh vertebrate blood, laid an average of 24 (±11) eggs whereas those fed on r-liq_diet laid on average 25 (±5) eggs per female. The best egg laying rates were achieved with r-liq_diet + P1 (hGLP1) (26 (±11) eggs per engorged female). Females fed on r-liq_diet + P5 (hCT) laid the lowest average number of eggs (n = 10) and this was in line with the significant reduction in percentage of egg development recorded (Fig. 2b). The data suggest that oogenesis and egg production was similar in mosquitoes fed a fresh-blood-free meal relative to mosquitoes fed on blood. In addition, the present results compared well with those of Sumba and colleagues^38^, in which the mean number of eggs oviposited by a laboratory-reared *A*. *gambiae* strain fed on the forearm of a human volunteer was of 22.6 ± 5.5 eggs/female.

### Mosquito fitness

**Figure 4:**
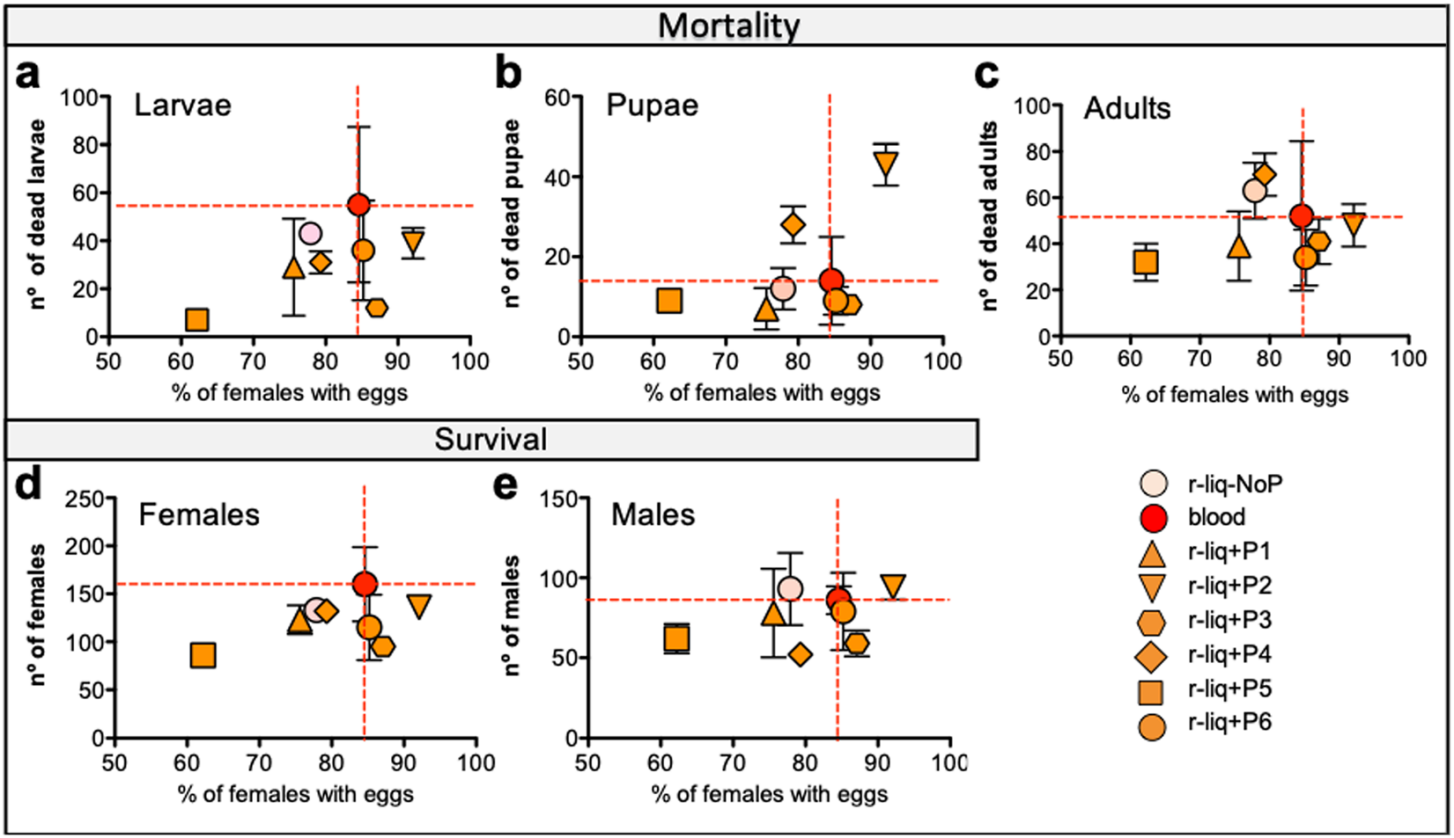
Effect of fresh-blood-free meals on mortality and survival. After egg laying, mosquitoes were followed through their life cycle until the last pupae emerged and data was analysed against the percentage of females with eggs. Progeny mortality for (**a**) Larvae, (**b**) Pupae and (**c**) Adults. Offspring’s progeny survival represented as (**d**) Number of females produced in the different diet groups and (**e**) Number of males produced in the different diet groups. Three independent experiments were performed, each consisting of 30 mosquitoes per diet (n = 90/diet). Vertical bars represent Standard Error of the mean (SE). No significant differences were observed between the blood-fed group and non-blood fed groups using an unpaired *t* test.

Mosquito fitness is a determinant factor for the success of a mosquito colony when they are released into the wild. Colonies from each diet were maintained under standard insectary conditions, and the life cycle was followed for one generation. Larvae, pupae and adult mortality were recorded. Offspring from females fed on r-liq_diet + P2 (hGLP2), P3 (hPTH) or P6 (hGABA) showed better performances (eg. lower rates of dead larvae and dead adults/egg number/female) (Fig. 4a–c) when compared to blood and other diets, however the total number of female progeny was slightly lower than in progeny of blood-fed females (Fig. 4d–e). A blood meal had the highest impact on larvae mortality (Fig. 4b), suggesting that stable highly nutritious artificial diets, without fresh blood, can reduce mortality improving mosquito rearing success. Variability (SE) was always higher in the blood group (Fig. 4 and Table 1) when compared with other diets and is probably a reflection of the variable composition of blood and emphasises the usefulness of fresh-blood-free diets.

Except for the r-liq_diet + P5 (hCT), the results obtained from the other dietary groups were similar to the blood fed colonies, showing a considerable advance over published studies (reviewed in^10^) in which artificial blood meals only showed relative success in *Aedes* mosquitoes, but were of limited or no-success when applied to *Anopheles* mosquitoes^39^. Recently a successful plasma-based artificial meal was described for *Anopheles* mosquitoes^40^ but lower feeding rates and estimated reproductive potential were obtained relative to the blood-fed control. Our results demonstrate that the r-liq_diet has a similar or superior effect on mosquito fitness relative to the standard vertebrate blood meal. Diet supplementation with vertebrate peptides affected different aspects of the female mosquito physiology and the progeny suggesting that they are also biologically active in the mosquito and interfere with reproduction and fitness. The fact that human calcitonin and salmon calcitonin reduced mosquito fecundity is intriguing and novel and suggests that crosstalk between the vertebrate peptides exist as they modify the physiology of the female mosquito. Human and salmon calcitonin are two small peptides of the same family that share 50% amino acid sequence identity and the fish peptide is a more potent activator of the human calcitonin receptor than the human peptide^41,42^. In vertebrates, calcitonin regulates mineral metabolism during pregnancy and lactation and peptide levels increase in the circulation. However, the reason why these peptides decrease mosquito fecundity is currently unknown. So far, no receptor that interacts with human or salmon CT has been identified in the malaria mosquito. Nonetheless, the identification of a molecule with the potential to decrease mosquito fecundity provides the basis for the design of more effective drugs in programs to reduce and control mosquitoes or other insect populations.

### Effect of the artificial diets on another Anopheles species oogenesis

As a proof of principal and to test the capacity of the formulated fresh-blood-free diets to trigger egg development in other *Anopheles* species r-liq_diet (with no peptide supplementation) was administrated to *A*. *stephensi* and *A*. *aquasalis* female mosquitoes using a similar procedure to that described above and egg production assessed 24 hours post-feeding. Developing eggs were identified in 70% to 93% of the *A*. *stephensi* females and 33% to 35% of the *A*. *aquasalis* females and the number of eggs per female was 53 ± 10 and 46 ± 27, respectively.

## Conclusion

The availability of a successful blood substitute diet will allow mass production of anautogenous mosquitoes without the need for costly animal care facilities or blood or plasma supply. The development of chemically well-defined artificial diet to provide a reliable and consistent nutrition to adult mosquitoes is a breakthrough for mass rearing of mosquitoes. Herein we described a formulated diet for anautogenous female *Anopheles* mosquitoes that mimicked a standardized vertebrate blood meal. The fresh-blood-free diet formulated stimulates oogenesis and egg production and has a similar or superior effect on mosquito fitness relative to a standard vertebrate blood in *Anopheles coluzzii* and we reveal it can also be used to feed other anopheline species. Supplementation of the blood-free diet with the vertebrate peptides that activate GPCRs regulating reproduction and metabolism reveals that they modify mosquito physiology. The putative mosquito GPCRs activated by the vertebrate peptides remain to be characterized. Of the tested human peptides, P2 (hGLP 2), had the most notable effect when it was introduced in the blood-free artificial diet and it significantly increased VTG expression, mosquito egg production and offspring fitness relative to blood-fed mosquitoes. P2 is a 33-amino acid peptide present in enteroendocrine L-cell and released in response to nutrient intake and it stimulates cell proliferation, inhibition of apoptosis and proteolysis in the small and large intestine in human^23,24^. It belongs to a family of peptides with functions in the gastrointestinal (GI) tract, carbohydrate metabolism and appetite regulation^43,44^. The effect of human or other vertebrate GLP2 peptides in invertebrates has not yet been explored. In contrast, P5 (hCT) had an inhibitory role on mosquito reproduction. The outcome of the study indicating potential co-evolution of protein-protein interactions and physiological systems between species that interact (eg. host – parasite/vector) remains to be further explored. The use of the artificial fresh-blood-free meal herein described for mosquito rearing will help reduce costs, effort and eliminate live animal use for mosquito culture opening-up new opportunities for vector-borne diseases control.

## Materials and Methods

Except otherwise indicated, all reactions were performed at room temperature (20 °C), reagents were purchased from Sigma-Aldrich Corporation (St. Louis, MO, USA), and female mosquitoes of *Anopheles coluzzii* (former *Anopheles gambiae* M form) Yaoundé strain were used. The peptides calcitonin (from human), calcitonin (from salmon), glucagon-like peptide 1 [1-37aa] (human, bovine, guinea pig, mouse, rat) trifluoroacetate salt, and glucagon-like peptide 2 [1-33aa] (human) ammonium acetate salt were purchased from Bachem (Germany). The peptides parathyroid hormone (from human), oxytocin (from human), kisspeptin 10 (from human), melatonin, and neuropeptide Y (from human, rat) were purchased from Tocris (Biogene, Spain).

### Mosquito rearing

All animal experiments were carried out in strict accordance with the Portuguese law and guidelines for the use of laboratory animals. The protocols used were approved by Direção-Geral de Veterinária, Ministério da Agricultura do Desenvolvimento Rural e das Pescas, Portugal (id approvals: 023351 and 023355). Mosquitoes were obtained from a laboratory colony of *A*. *coluzzii* (Yaoundé line) and *Anopheles stephensi*. *Anopheles aquasalis* were obtained from a colony at the Entomology Department Insectary of the *Fundação de Medicina Tropical Dr Heitor Vieira Dourado* (FMT-HVD) (id approvals: CEUA 01/2013), that were derived from a colony established in 1995^45^. Mosquitoes were maintained under standard insectary conditions of 26 ± 1 °C, 75% humidity and a 12 h:12 h light:dark cycle. Adult mosquitoes were fed on 10% glucose solution *ad libitum* until the day before feeding trials.

### Feeding of mosquitoes

Female CD1 mice (*Mus musculus*), obtained from the IHMT Animal house, were intraperitoneally anesthetized with 20% Imalgène 1000 (Merial, Portugal) and 10% Rompun (Bayer, Portugal). Female mosquitoes fed directly on healthy female CD1 mice for 30–45 min, with regular monitoring to verify that mice were anesthetized. Unfed mosquitoes were removed and fully engorged mosquitoes were kept at 26 ± 1 °C, 75% humidity. When needed, a cardiac puncture was performed on anesthetized mouse to collect 1 mL of blood for artificial blood feeding assays.

### Fluorescence confocal microscopy

The ovaries of *A*. *coluzzii* female mosquitoes were collected twenty-four hours after feeding on blood or on supplemented liquid diet and fixed for 15 min with 4% v/v paraformaldehyde (Alfa Aesar, Massachusetts, USA) in PBS. Samples were washed twice with 0.5% Triton in PBS and incubated with 250 µM Nile Red (MP Biomedicals, USA) for one hour in the dark. Samples were washed twice with 0.5% Triton in PBS and the slides were mounted by using Vectashield with dapi (Vector Laboratories, USA) and analyzed using a Leica TCS SP5 laser scanning confocal microscope (20x, 40x, and 60x objectives).

### RNA extractions & cDNA synthesis

Total RNA from female fatbodies was isolated using the TRI Reagent protocol and treated with 1 U DNase for 1 min according to the manufacturer’s instructions to eliminate genomic DNA contamination. DNase I treated total RNA (1,5 µg) was denatured at 70 °C for 10 min, quenched on ice for 5 min and used for cDNA synthesis in a 20 µl final reaction volume containing 10 µL of 2 × RT buffer mix, 1 µL of 20 × RT enzyme mix (Thermofisher, Alfagene, Portugal), and nuclease-free water. cDNA was synthesized for 60 min at 37 °C followed by 5 min at 95 °C to stop the reaction and hold at 4 °C. The quality and quantity of the cDNA produced was assessed by PCR amplification of the ribosomal protein S7 unit using the following protocol: 95 °C for 3 min; 35 cycles of 95 °C for 30 sec, 60 °C for 30 sec, 72 °C for 30 sec, followed by 72 °C for 5 minutes^46^. PCR reactions were carried out for a 10 µl final reaction volume containing 1.5 mM MgCl_2_ (Thermo Scientific, Alfagene, Portugal), 0.2 mM dNTPs (GE Healthcare, Spain), 0.25 µM of each gene specific primer pair and 0.5 U of DreamTaq DNA Polymerase (5 U/µl, Thermo Scientific, Alfagene, Portugal) and the amplification products analysed on agarose electrophoresis gel.

### Quantitative Polymerase Chain Reaction

Quantitative Real-time PCR (q-RT-PCR) analysis was used to quantify the expression of Vitellogenin-1 precursor (Vg), a protein biomarker of mosquito oogenesis initiation. Vg expression levels in the fatbodies were quantified using the ΔΔCT method a) after a blood meal, b) after microinjection with the different peptides, and c) after being fed on the various artificial diets. Primers used are described in Table S2 (Supplementary Information). Briefly, cDNA samples were diluted (1:10 or 1:5) with ultrapure water prior to use as a template in q-RT-PCR and reactions were performed in triplicate (<5% variation between replicates) using a CFX Connect Real-Time PCR Detection System (Bio-Rad, Portugal) for 96-well microplates (Bio-Rad, Portugal). Analyses were performed in 20 µl final reaction volume with 300 nM of forward and reverse primer, SsoFast EvaGreen supermix (Bio-Rad, Portugal) and 2 µl of the diluted cDNA template. Optimized cycling conditions consisted of 95 °C for 30 sec, followed by 45 cycles of 95 °C for 5 sec and the appropriate annealing temperature for each primer pair for 10 sec. PCR reactions included a standard curve, melting curves were performed to detect primer dimers and negative control reactions were included to assess for genomic contamination. PCR reaction efficiencies and r^2^ (coefficient of determination) were calculated for each target gene and transcript expression was normalized using ribosomal S7 subunit as reference gene.

### Peptide microinjections

Two-day-old female *A*. *coluzzii* mosquitoes were cold-anaesthetized and injected intrathoraxically with 69 nL of 10 µM peptide. For each experiment, a control group injected with PBS was included. Injections were performed using a microinjection system (Nanoject; Drummond Scientific). The complete list of the peptides used and respective abbreviations are presented on Supplementary Information, Table S1.

### Artificial feeding

Mosquitoes (n = 30 approximately) were kept inside paper cups covered with a net. Each cup had a glass feeder on top connected to 2 plastic tubes for water inlet and outlet and temperature within the multiple cylindrical water-jacked plastic was kept at 37.5 °C by a constant water flow supply. Parafilm® was stretched across the mouth of the feeder and 1 mL of a pre-warmed meal was pipetted into the glass feeder. Peptides were tested at a 10 µM final concentration. Mosquitoes were allowed to feed for 60 minutes. Unfed mosquitoes were removed and fully engorged female mosquitoes were kept at 26 ± 1 °C under 75% humidity.

### Liquid diet recipe

The i-liq_diet consisted on Dulbecco’s modified Eagle’s medium (high glucose with L-glutamine from Lonza, 12–604 F). The formulation of the enriched artificial meal (r-liquid diet) is listed on Supplementary Information, Table S3. ATP, cholesterol and BSA were purchased from Sigma-Aldrich Corporation (A6419, C4951 and A7906 respectively). For each experiment, diet formulae were freshly prepared from stock solutions.

### Tissue collection

Fatbodies were collected from pools of 30–35 mosquitoes. Tissues were dissected, transferred to RNAlater (Ambion, Alfagene, Portugal) and stored at −20 °C until RNA extraction. For the blood meal assays, mosquito fatbodies were collected at different time points (3, 6, 12, 24, 28, and 32 hours post blood meal). Fatbodies from females feed on 10% glucose at the same time points were used as controls. For the peptides screening experiment and artificial diet assays, mosquito fatbodies were dissected 24 h post-injection and 24 h post-feeding, respectively.

### Fecundity, fertility and adult, pupae and larvae development and mortality

Experimental feeding of mosquitoes using blood, i-liquid diet, r-liquid diet (Supplementary Information, Table S3) or r-liquid diet supplemented with blood peptides (Supplementary Information, Table S1) was performed as described above. As a proxy of feeding success, the number of mosquitoes that were fully engorged was recorded and the percentage of fed mosquitoes determined. To determine the number of eggs per female, at 48 h post-feed, mosquitoes were dissected, the ovaries collected and the number of eggs per female determined under a hand held magnifying glass. For egg laying and mosquito development, 30 fully engorged females were placed in individual cages (20 × 20 × 20 cm) with humidified filter paper for egg laying (30 females/replicate, 3 replicates/diet). Eggs were counted 48 and 72 h post-feed under a hand held magnifying glass. The total number of eggs/female was registered.

Eggs were collected into a water tray (23 × 15 × 6 cm) and mosquito development was followed until all pupae have emerged, for each diet. After hatching, a strict similar larval feeding regime was applied to all trays, using grounded fish food (1:1 ASTRA pond-fish-sticks and TetraMin flacs). Trays were fed approximately 13 mg every day. Water levels were kept constant throughout the experiment by addition of dH_2_O. Pupae and larvae (L3 and L4) mortality was checked daily and dead stages were removed. Although there was abundant food, the dead larvae could have been cannibalized. As the larvae resulting from females fed with different diets were in similar conditions, we assume that if it occurs, cannibalism would be similar in all groups. The number of male and female adults for each diet was recorded by the end of the experiment when all pupae have developed into adult mosquitoes. Dead adults (females and males) were counted and removed daily. Adults were maintained on water with 10% glucose solution *ad libitum* until the day they died. The experiment ended when all pupae developed into adult mosquitoes in all diets. Three independent replicates were performed for each diet.

### Statistical analysis

Data are presented as the mean ± standard deviation of at least three independent experiments (except where otherwise indicated), and the corresponding standard deviations in histograms are represented by error bars. The Student’s t test was used to compare independent groups when data followed a Gaussian distribution, and differences were considered significant when P ≤ 0.05. The Fisher’s exact test was used to compare the differences on proportions among distinct diet-fed groups. The statistical analysis was performed on GraphPad Prism6 software.

## Electronic supplementary material

Supplementary Information
