# Supplementary material for: Fresh-blood-free diet for rearing malaria mosquito vectors
Source: Sci Rep. 2018 Dec 13;8:17807. doi: 10.1038/s41598-018-35886-3 (PMC6292920; doi:10.1038/s41598-018-35886-3)
Supplement: Supplementary file 1 — Supplementary Information [file 41598_2018_35886_MOESM1_ESM.docx]

Supplementary Information

**Fresh-blood-free diet for rearing malaria mosquito vectors**

Joana Marques, João C. R. Cardoso, Rute C. Felix, Rosa A. G. Santana, Maria das Graças V. Barbosa Guerra, Deborah Power, Henrique Silveira*

* Corresponding author: hsilveira@ihmt.unl.pt

**This file includes:**

Fig. S1

Tables S1 to S3


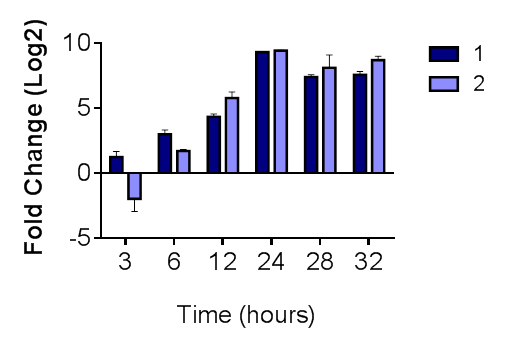


Fig. S1. Time frame expression of vitellogenin precursor. Expression levels were analysed at 3, 6, 12, 24, 28, and 32 hours post-feeding and is represented as Log2 of 3 biological replicates. Relative expression was determined by the ΔΔCT method of q-RT-PCR with mosquitoes fed on 10% glucose. A total of 2 independent experiments were performed (series 1 and series 2). We studied the Vg expression by q-RT-PCR (Figure S1) as an indicator for reproduction activation since it is the most highly expressed gene involved in vitellogenesis. Mosquito fatbodies were collected at different time points (3, 6, 12, 24, 28, and 32 hours post-blood meal) and a control group was fed on 10 % glucose. For each time point we used 30 female mosquitoes.

Table S1. List of the peptides used and abbreviations

| **Peptide** | **Abbreviation** |
| --- | --- |
| Glucagon-like peptide 1 | P1 |
| Glucagon-like peptide 2 | P2 |
| Parathyroid hormone | P3 |
| Salmon calcitonin | P4 |
| Human calcitonin | P5 |
| ɣ-aminobutyric acid | P6 |
| Salmon Luteinizing Hormone Releasing Hormone | P7 |
| Human Luteinizing Hormone Releasing Hormone | P8 |
| Galanin | P9 |
| Vasoactive intestinal peptide | P10 |
| Glutamate | P11 |
| Oxytocin | P12 |
| Kisspeptin | P13 |
| Neuropeptide Y | P14 |
| Melatonin | P15 |
| Corticotropin-releasing factor | P16 |
| Combination of peptides 1 to 6 | P Mix |

Table S2. Primers used for q-RT-PCR reactions. Amplification was performed at 64 ºC for S7 and 60 ºC for Vg

| Target |  | Sequence 5’-3’ |
| --- | --- | --- |
| S7 (AGAP010592) | Forward | GAGGTGGTCGGTATCC |
|  | Reverse | CGATGGTGGTTTATCC |
| Vg (AGAP004203) | Forward | ACGAAAACCATGACCGCTCT |
|  | Reverse | CTTGGGACGGATCACCAAAT |

Table S3. Composition of r-liquid diet

| **Components** | **g/L** |
| --- | --- |
| Adenosine Triphosphate | 0.55 |
| Bovine Serum Albumin | 200 |
| Calcium chloride anhydrous | 0.2 |
| Cholesterol | 1 |
| Choline chloride | 4.00E-03 |
| D-calcium pantothenate (vitamin B5) | 4.00E-03 |
| D-glucose anhydrous | 4.5 |
| Ferric nitrate nonahydrate | 1.00E-04 |
| Folic acid | 4.00E-03 |
| Glycine | 0.03 |
| I-inositol | 7.00E-03 |
| L-arginine monohydrochloride | 0.084 |
| L-cystine dihydrochloride | 0.063 |
| L-glutamine | 0.584 |
| L-histidine monohydrochloride monohydrate | 0.042 |
| L-isoleucine | 0.105 |
| L-leucine | 0.105 |
| L-lysine monohydrochloride | 0.146 |
| L-methionine | 0.03 |
| L-phenylalanine | 0.066 |
| L-serine | 0.042 |
| L-threonine | 0.095 |
| L-tryptophan | 0.016 |
| L-tyrosine disodium salt dihydrate | 0.104 |
| L-valine | 0.094 |
| Magnesium sulfate anhydrous | 0.098 |
| Niacinamide (nicotinamide) | 4.00E-03 |
| Phenol red | 0.015 |
| Potassium chloride | 0.4 |
| Pyridoxine Monohydrochloride | 4.00E-03 |
| Pyruvic acid sodium salt | 0.011 |
| Riboflavin (vitamin B2) | 4.00E-04 |
| Sodium bicarbonate | 3.7 |
| Sodium chloride | 6.4 |
| Sodium phosphate monobasic anhydrous | 0.109 |
| Thiamine amonohydrochloride (vitamin B1) | 4.00E-03 |
